# A Fatal Case of *Klebsiella pneumoniae* Mycotic Aneurysm

**DOI:** 10.1155/2011/498545

**Published:** 2011-11-29

**Authors:** Chien-Ming Chao, Kun-Kuang Lee, Chia-Sheng Wang, Ping-Jen Chen, Tsung-Chih Yeh

**Affiliations:** ^1^Department of Surgery, Chi Mei Medical Center, Liouying, Tainan 736, Taiwan; ^2^Department of Intensive Care Medicine, Chi Mei Medical Center, Liouying, Tainan 736, Taiwan

## Abstract

Mycotic aneurysm is a serious clinical condition with significant morbidity and mortality. *Staphylococcus aureus* and *Salmonella* species are the most common causative pathogens. *Klebsiella pneumoniae* was rarely reported as a possible pathogen causing mycotic aneurysm; therefore, we describe a *K. pneumoniae*-related fatal bacteremia mycotic aneurysm in a patient in spite of appropriate antimicrobial agents and surgical management.

## 1. Introduction

Although mycotic aneurysms infrequent, which only account for 2.5% of all aneurysms, and 1.8% of all thoracoabdominal aortic aneurysms [1–3], it is a serious clinical condition with significant morbidity and mortality. The majority of mycotic aneurysms are caused by bacteria, and *Staphylococcus aureus* and *Salmonella* species are the most common causative pathogens [1, 4]. In addition, other bacteria including *Pseudomonas* spp., *Campylobacter fetus*, *Streptococcus* spp., *Clostridium *spp., and *Corynebacterium* spp. have been reported as possible pathogens causing mycotic aneurysm [4]. Herein, we describe a *K. pneumoniae* causing fatal bacteremia mycotic aneurysm in a patient. 

## 2. Case Report

**Figure 1:**
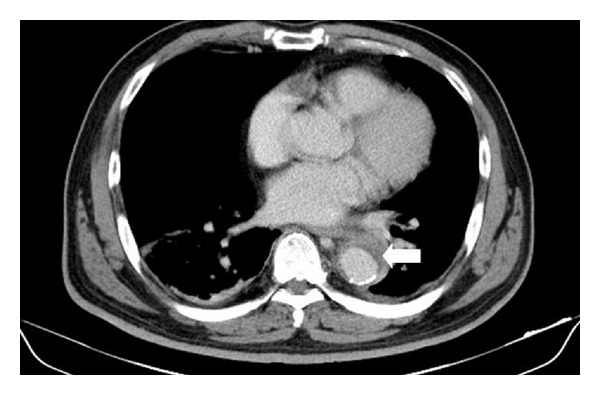
Computed tomography of chest showed aortic intramural hematoma (arrow).

**Figure 2:**
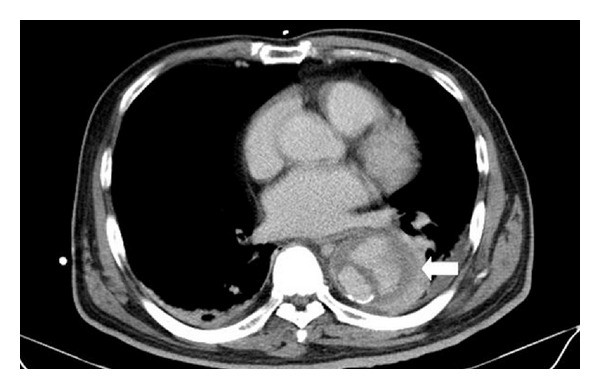
Computed tomography of chest showed progressive dilated aorta and enlarged intramural hematoma (arrow).

A 47-year-old man with a history of hypertension presented with fever, dyspnea, and chest discomfort for one day. He denied any other medical history, including HIV infection. On arrival in the emergency department, his body temperature was 38°C, and blood pressure was 207/125 mm Hg. Physical examination was unremarkable. The laboratory findings were as follows: white blood cell 14300/mm^3^ with predominance of neutrophils (89.8%) and C-reactive protein 213 mg/L (normal reference < 6 mg/L). Cardiac enzyme, arterial blood gas analysis, renal function, and electrolytes were within normal range. Chest X-ray did not show pneumonia patch, and urinalysis did not reveal pyuria. Computed tomography (CT) of chest and abdomen showed an aortic intramural hematoma from the arch to suprarenal level (Figure 1). Intravenous ceftriaxone (2 g every 12 hours) was administered under the impression of infected aortic aneurysm after collection of two sets of blood culture. Two days later, cultures of blood grew *K. pneumoniae* which is only resistant to ampicillin. Because he continued having intermittent pyrexia, antibiotic was switched to ceftazidime (2 g every 8 hours) and gentamicin (80 mg every 8 hours). Thereafter, the patient's fever gradually subsided, and repeat blood cultures were negative. On the 20th day, sudden onset of fever (39°C) recurred, and hypotension developed. Repeat CT showed progressive dilated aorta and enlarged intramural hematoma (Figure 2). Emergency operation with aortic grafting was performed, but the patient's condition gradually deteriorated, and the patient died eight days after surgery. The repeated blood culture on hospital day 20 and the specimen of excised aorta all grew *K. pneumoniae. *All of the *K. pneumoniae* isolates have the same antibioticgram, which is only resistant to ampicillin. The pathology report disclosed atherosclerosis, necrotizing inflammation, organized hematoma, and bacterial clumps in the aortic wall, which are compatible with mycotic aneurysm. Finally, the diagnosis of *K. pneumonia*-related mycotic aneurysm was confirmed. 

## 3. Discussion

*K. pneumoniae* is a member of Enterobacteriaceae and is the normal flora of the human mouth and intestine. The clinical manifestations of *K. pneumoniae* infection are protean and include pneumonia, bacteremia, intra-abdominal infection, skin/soft tissue infection, and osteomyelitis. However, cases of *K. pneumoniae* mycotic aneurysm were limited, and diabetes mellitus is the most common risk factor, which present in most of the reported cases [5–10]. In the present case, he did not have diabetes mellitus, which is in contrast to the previous reports [5–10].

The mortality of mycotic aneurysm remains high, and early diagnosis is important since catastrophic hemorrhage or uncontrolled sepsis may occur without prompt medical and surgical management. Although *K. pneumonia* was a rare pathogen causing mycotic aneurysm, appropriate antibiotic should be given according to the susceptibility test, and surgical intervention may be warranted once the diagnosis of *K. pneumonia* infection is established. In our case, emergent surgery was performed, while rupture of aneurysm and further uncontrolled sepsis led to the fatal outcome. This should suggest that close monitor and evaluation of the clinical condition and image might be needed. If we can early detect the progression of mycotic aneurysm after medical treatment failure, prompt surgery may save the life.

There is one major limitation in this study. Because there is no available isolate for further study, we cannot evaluate the microbiological characteristics, such as the hypermucoviscosity, capsular serotype, the presence of* rmpA* or genetic relatedness of all of the *K. pneumoniae* isolates to clarify the molecular feature of these causative isolates and further exclude the possibility of superinfection. However, based on our finding about the same antibioticgram of all isolates, they were supposed to have the same strain.

In conclusion, the demonstrated case expands the spectrum of infection caused by *K. pneumoniae* and raises the possibility of *K. pneumoniae* as one of the causes of mycotic aneurysms in immunocompetent patient, even in patients without diabetes. Prompt diagnosis and antibiotic therapy with or without surgery are crucial for better outcome.
